# A delayed diagnosis of a case with multiple system atrophy

**DOI:** 10.1002/alz.093559

**Published:** 2025-01-03

**Authors:** Mohamed Elkasaby, Elliott Hayden, Brian Appleby

**Affiliations:** ^1^ University Hospitals Cleveland Medical Center, Cleveland, OH USA; ^2^ Case Western Reserve University, Cleveland, OH USA; ^3^ University Hospitals Cleveland Medical Canter, Cleveland, OH USA

## Abstract

**Background:**

A patient presented to movement disorder clinic with cognitive complaints, imbalance and prior diagnosis of NPH. The patient underwent ventriculoperitoneal shunt in the past with minimal improvement, a detailed history is suggestive of REM sleep behavioral disorder, autonomic dysfunction including orthostatic hypotension and urinary incontinence.

**Method:**

Clinical evaluation was notable for bradykinesia, rigidity, truncal and cervical dystonia, shuffling steps, reduced arm swing bilaterally and pink, dusky skin of both hands.

**Result:**

DaTscan showed reduced uptake of dopamine transporter.

**Conclusion:**

Evaluation at movement disorder clinic helps early diagnosis of neurodegenerative disorders with subtle findings which allows patients to benefit from available treatments and participation in appropriate clinical trials.

